# Variability in the prescription of drugs with uncertain effectiveness. The case of SYSADOA in the Basque Country

**DOI:** 10.3205/hta000130

**Published:** 2018-11-05

**Authors:** Nora Ibargoyen-Roteta, Maider Mateos del Pino, Iñaki Gutiérrez-Ibarluzea, Gaizka Benguria-Arrate, Diego Rada-Fernández de Jauregui, Cristina Domingo-Rico, Iratxe Regidor Fuentes, Roberto González Santisteban, María Armendáriz Cuñado, Nekane Jaio Atela

**Affiliations:** 1 Osteba (Basque Office for Health Technology Assessment), Ministry for Health, Basque Government, Vitoria-Gasteiz, Spain; 2 Health Research and Studies Unit, Ministry for Health, Basque Government, Vitoria-Gasteiz, Spain; 3 Department of Preventive Medicine and Public Health, University of the Basque Country/Euskal Herriko Unibertsitatea (UPV/EHU), Vitoria-Gasteiz, Spain; 4 Medical Directorate of the Interior Care Setting, Osakidetza, Basque Health Service, Amorebieta, Spain; 5 Quality and Research Unit, Interior Care Setting, Osakidetza, Basque Health Service, Amorebieta, Spain; 6 Interior Care Setting, Osakidetza, Basque Health Service, Amorebieta, Spain; 7 Pharmaceutical Unit, Barrualde-Galdakao Integrated Health Services Setting, Osakidetza, Basque Health Service, Amorebieta, Spain

**Keywords:** de-prescription, disinvestment, SYSADOA, chondroitin sulfate, diacerein, glucosamine, osteoarthritis

## Abstract

**Background: **The majority of clinical practice guidelines do not recommend the use of SYSADOA (Symptomatic Slow Action Drugs for Osteoarthritis) for the treatment of osteoarthritis because of the lack of evidence or uncertainty around their efficacy. Nevertheless, the Spanish Public Health Service continues funding these drugs.

**Aim:** The aim of this study is to describe the prescription status of SYSADOA in the primary care units of the Basque Country during 2011; to determine if variability exists among them; and to examine if the variability could be explained by the health care region each PC unit belongs to.

**Methods:** Prescription data for SYSADOA during 2011 was obtained from the Basque Ministry for Health. In the Basque Country, primary care is divided into seven regions, each region consisting of several primary care units, which were used as the unit of analysis. Defined daily doses (DDD) per 1,000 inhabitant-days (DHD) were calculated. Data were standardized by sex and age using the total population of the Basque Country as the reference population. Small area statistics were calculated (extremal quotient, coefficient of variation and systematic component of variation). The influence of the region to which primary care units belonged was also analysed. R software (version R-2.15.0) was used for the analysis.

**Results:** SYSADOA prescription during 2011 accounted for an expense of 4.5 million euros for the Basque Health Service. The crude rate of consumption of SYSADOA was 7.81 DDD per 1,000 inhabitant-days. The obtained external quotient was 13.67. The prescription of SYSADOA of the primary care units located in the 95th percentile was six times higher than the ones located in the 5th percentile. The region to which units belonged accounted for 57% of the observed variability.

**Discussion:** The uncertainty around these drugs could be reflected in the existing variability of their prescription level. The analysis of the variability in the prescription of drugs with no demonstrated efficacy could help in allocating resources into other services or health technologies supported by evidence, thereby contributing to the improvement of health outcomes.

## Introduction

In the last two decades, the prescription of a group of drugs approved specifically for the treatment of osteoarthritis has increased and, along with it, pharmaceutical expenditure [1]. They are named SYSADOA (Symptomatic Slow Action Drugs for Osteoarthritis) or slow-acting drugs.

The following SYSADOA are funded by the Spanish health care system: glucosamine sulfate, chondroitin sulfate and diacerein. It is estimated that the consumption of SYSADOA has increased from 2.2 Standardized Defined Daily Doses (DDD) per 1,000 inhabitants (DHD) in 2000 to 11.5 DHD in 2012 (a 423% increase) [2].

In some regions of the Spanish health care system, the outpatient publicly funded consumption of oral SYSADOA doubled in less than 5 years, from 150,000 DDD per million inhabitants in January 2005 to more than 300,000 DDD in December 2009. The economic impact of the use of this group of drugs in these regions is also very important: during the year 2009, the oral SYSADOA accounted for a spending of 1.7 million euros per million inhabitants (official prescription billing) [3].

In recent years, glucosamine sales have grown dramatically around the world, reaching 2,000 million US dollars in 2008 (an increase of 60% compared to sales in 2003) [4]. In addition, it is expected that the sales of these drugs will continue growing, mostly because the population is getting older in high-income countries, and osteoarthritis is a chronic condition. 

Despite the numerous studies that have been published on the controversy regarding oral administration of SYSADOA and their efficacy in the treatment of osteoarthritis [5], [6], [7], [8], [9], [10], [11], [12], the current evidence supporting their effectiveness – both symptomatic and structural – is inconsistent and does not recommend their use [5], [6], [10], [12].

The results of previous studies are contradictory, and those reporting better results have small sample sizes and are of questionable quality [5], [9], [13], [14]. The inconsistencies are related to the use of different scales of measurement of pain, different follow-up periods, the inclusion of patients with different levels of progression of the disease, different age or sex, the use of different doses or different formulations (for example glucosamine sulfate or hydrochloride). Moreover, the existence of conflicts of interest in many of these studies and the sensitivity of the results did not promote trust in the obtained final outcomes [9].

Interestingly, in a previous report it was stated that those guidelines on the management of osteoarthritis obtaining a higher AGREE II score for the “rigor of methodology” domain did not recommend the prescription of any single or combined SYSADOA for the management of osteoarthritis, and that Clinical Practice Guidelines (CPG) recommending their use have important methodological weaknesses [15].

Furthermore, and in line with CPGs, various committees of drug evaluation, as those of the Basque Country and the region of Navarre, have published evaluation reports which concluded that the combination of chondroitin sulfate and glucosamine does not imply additional therapeutic value based on current evidence [16], [17]. 

Studies of variability in population-based patterns of prescription and drug use are a useful tool for reporting measures and differences in pharmacological prescription of dubious or no effectiveness. This analysis could lead to tailored actions of disinvestment in clinical practice in those areas in which high prescription rates are not duly justified. In this case, differences in rates of prescription of so called “chondroprotectors” could signal a differential management of osteoarthritis in different health areas [18].

The interest around variability in the prescription of drugs is due to the possibility that this variability hides a misuse of resources, either through over-use or under-use, but which, in either case, would mean an inadequate quality of care [19], [20].

Therefore, the aim of this study was to determine the rate of prescription of SYSADOA in the Basque Country in 2011 and analyse the variability in the prescription of these drugs by primary care health units, with the health care region as a possible explanatory factor of this variability.

## Methods

We conducted a cross-sectional ecologic study on the indication and prescription of SYSADOA in the treatment of osteoarthritis during the year 2011, which included an analysis of the variability observed among primary care settings (PCSs). We also studied the association between the level of prescription of these drugs and the health region PCSs belonged to.

Since the PCSs were taken as the unit of analysis, we studied 132 settings that give coverage to the entire population of the Autonomous Community of the Basque Country.

We considered three active ingredients used in the treatment of osteoarthritis (glucosamine sulfate, chondroitin sulfate and diacerein), as well as a composite that combines those first two (even if it was not marketed for use before July 2011). The ATC-WHO codes for each active ingredient were: M01AX05, M01AX25, M01AX21 and M01CX. 

The number of people belonging to each area, that is, the denominator of the rate of prescription, was obtained from population data systems (Individual Health Card on December 31, 2011). Furthermore, information on prescription was anonymously extracted from the databases by personnel from the Directorate of Pharmacy of the Ministry for Health of the Basque Government. Since these databases are fed from billing prescriptions, the data refer to prescriptions of the Basque Health System. Readers should bear in mind that the Basque Health System has universal health coverage and that 93% of prescriptions are publicly funded. The data-recording system does not include the diagnosis associated with every prescription. According to market approval on indications for SYSADOA, we assumed that the total number of prescriptions of each active ingredient has been made for preventive or curative treatment of osteoarthritis.

The number of DDD (using the ATC/DDD system: for glucosamine sulfate, the code is M01AX05) was calculated for each PCS per 1,000 inhabitants per day (DHD) and, using the direct method, the age- and sex-standardized rate of consumption were estimated. For this purpose, the total population of the Basque Country was taken as the reference population. This calculation was performed both by individual active ingredient and in an aggregate manner. With these rates, a descriptive analysis of the prescription was conducted through the percentiles and dot-plots in natural scale and logarithmic scale of mean 0. The latter representation allows a more adequate comparison of the variation between the different active ingredients, avoiding that those presenting lower rates are accumulated in the base of the graphic showing minor variation with no outliers.

Lastly, we studied the variability between PCSs calculating the parameters employed in small-area analysis: extremal quotient, coefficient of variation and systematic component of variation. All these parameters were obtained from the rates in areas located between percentiles 5 (P_5_) and 95 (P_95_), in order to avoid the possible influence of the extreme values.

Finally, an analysis of variance was made to assess whether membership of the PCSs to one region or another influences the rates of consumption of these drugs, i.e., if the PCSs belonging to a specific region behave similarly or differently from the PCSs of other regions with regard to SYSADOA.

All analyses were conducted using the R statistical software (version R-2.15.0). The operating system used was Microsoft Windows.

## Results

During 2011, a total of 6,786,493 DDD of SYSADOA corresponding to prescriptions issued by 132 units of primary care (Table 1 (Tab. 1)) were prescribed in the Basque Country. This amount resulted in a crude consumption rate of 7.81 DDD per 1,000 inhabitants per day, with chondroitin sulfate being the most dispensed drug (3.61 DHD) and the chondroitin sulfate and glucosamine combination being less frequently dispensed (0.13 DHD).

The prescription costs by the PCSs were 3,393,036 euros – however, this expenditure increased to 4.5 million euros if we took into account the prescriptions issued by hospitals, private centers and private professionals that could not be assigned to age-group and sex (9,188,174 DDD).

**Figure 1:**
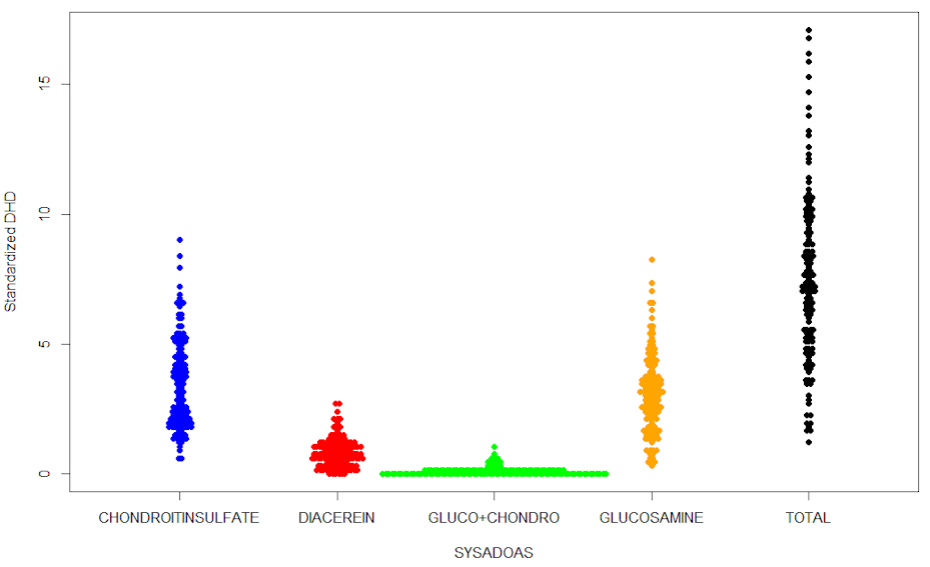
Standardized rates classified by drug. Each point represents a primary care setting.

Table 1 (Tab. 1) shows the distribution of consumption rates standardized by age and sex, for each of the four drugs separately and in aggregate. The areas reported aggregated rates ranging between 1.25 and 17.09 DHD. However, the values of the 5th and 95th percentiles allowed us to observe that there were outliers in both tails of the distribution. This phenomenon appeared in all the study drugs. The magnitude of rates and their distribution by areas was similar for chondroitin sulfate and glucosamine, whose medians were 3.22 and 3.11 DHD, respectively. Far from these amounts we found that diacerein (median=0.78) and the combination of chondroitin sulfate and glucosamine turned out to be the least consumed compounds (mean=0.07), as already indicated by its crude rate. However, it has to be noted that the combination of drugs was not marketed for use before July 2011. All these results are shown in Figure 1 (Fig. 1).

**Figure 2:**
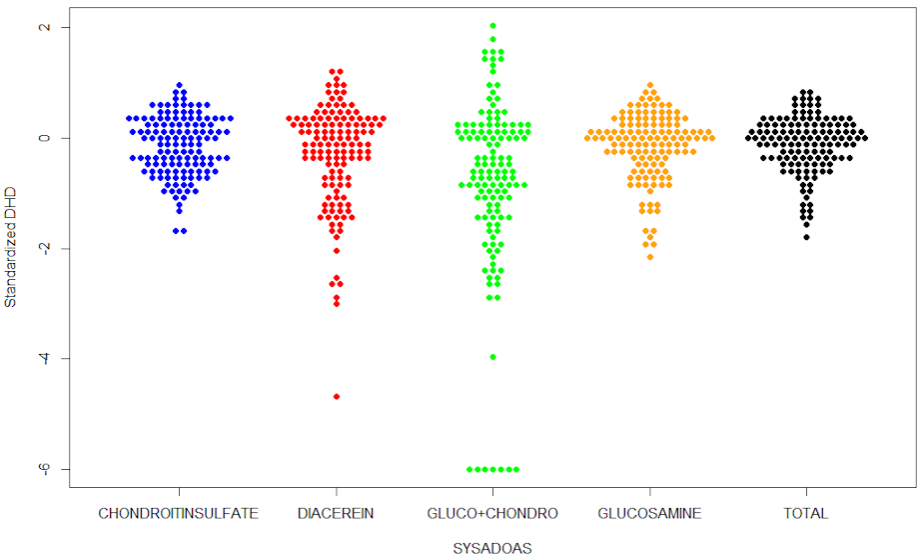
Standardized rates on a logarithmic scale of average 0 classified by drug. Each point represents a primary care setting.

In addition, Table 1 (Tab. 1) shows the values obtained for the statistical variation. The overall extremal quotient (EQ) indicates that the PCSs in the 95th percentile consumed 6 times more than the ones located in the 5th percentile. The EQ values by active ingredient were also high, being especially important in the case of the combination of chondroitin sulfate and glucosamine. However, such values are justified by the existence of small areas in which the prescription of this drug was in effect zero. The coefficients of variation pointed in the same direction and varied between 0.39 for glucosamine and 0.92 for the combination of drugs. The value of this parameter when considering the four drugs altogether was reduced to 0.35. Figure 2 (Fig. 2) allows the comparison of the variability between the different drugs, showing the slight reduction of variability when aggregated.

With regard to the systematic variation (not expected by chance), the systematic component of variation had a value of 0.19 for total prescription. When we analyzed data by drug, we detected higher variations ranging from 0.35 for chondroitin sulfate to 0.97 for the combination of this drug with glucosamine. In either case, these data indicate that the systematic variation was high.

**Figure 3:**
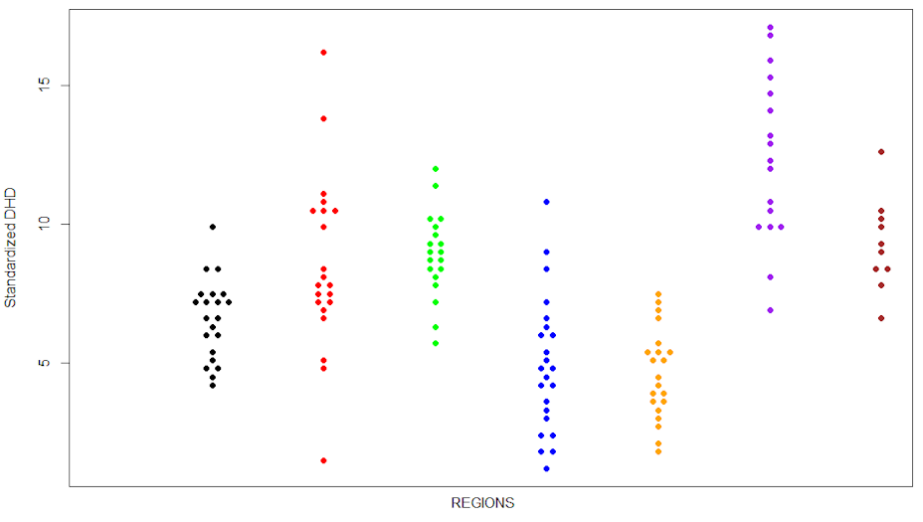
Standardized rates classified by region. Each point represents a primary care setting.

Figure 3 (Fig. 3) represents prescription rates by PCSs according to the region in which they are located. It is shown that belonging to one or another region influenced the consumption of SYSADOA. The results obtained through the analysis of variance (Table 1 (Tab. 1)) corroborated that the factor health region explains much of the variance between areas. In fact, the aggregated coefficient of determination was 0.57, and differentiating by active ingredient, this parameter varied between 0.42 and 0.65.

## Discussion

The results of this study show that in PCSs in the Basque country, a total of 6,786,493 SYSADOA DDD were prescribed in 2011, resulting in an expenditure of 3.4 million euros for our public health care system.

None of the guidelines that address the treatment of osteoarthritis make a recommendation with a level of sufficient evidence to justify such a high prescription rate and such high spending on these drugs, either as an active ingredient or in combination [15]. In fact, the committee of evaluation of new drugs of the Basque Country published an assessment report in 2012 [16] which was disseminated to all physicians in the Basque Country. This report emphasized that the combination of chondroitin sulfate and glucosamine has not been shown to be superior to placebo in the symptomatic treatment of osteoarthritis of the knee and that neither the chondroitin sulfate nor the glucosamine prescribed as monotherapy had proven to be superior to placebo in the control of pain and in the reduction of radiological progression. 

Our data showed that the most prescribed drug was chondroitin sulfate (3.61 DHD), followed by glucosamine (3.20 DHD). The combination of chondroitin sulfate with glucosamine (0.13 DHD) was dispensed least frequently, but this is consistent with market access authorisation, since the combination was not marketed in Spain before July 2011 [16].

The extreme quotient between the PCSs that lie in the 5th and 95th percentiles is 6, which means that the PCSs in the 95th percentile consumed 6 times more SYSADOA than the ones located in the 5th percentile. In our study we calculated prescription rates standardized by age and sex, so the differences in prescription rates cannot be explained by differences in the demographic characteristics or prevalence of osteoarthritis among PCSs. A more plausible explanation is that the variability results from different medical decisions in similar situations. According to the so-called hypothesis of uncertainty, variability in the treatment of similar patients is caused by insufficient information on the efficacy of treatments in some specific situations, and by a poor implementation of the available evidence into the real-world clinical practice [21].

One of the criteria that has also been identified as a cause of geographic health care variation is the existence of differences in the perception of the clinical value of interventions [22]. In the case of SYSADOA, we found the following:

1. Even if there is no clear evidence for these drugs’ effectiveness, they are safe, since no major side effects have been reported.
2. There is no efficacious treatment other than painkillers for symptomatic osteoarthritis in those cases in which surgical treatment is not indicated.

These circumstances could lead to different points of views regarding prescription on the part of clinicians.

In our study, we noted that one of the factors that influence the prescription of SYSADOA considerably was the health region the PC unit belonged to. Indeed, the region factor explains much of the variance between areas, which could be a result of the prescription induced by certain specialized care centers that are responsible for the first indication and prescription. However, it would be beyond the scope of our analysis to explore this in more detail. Gabbay and May pointed out that: “Clinicians rarely accessed and used explicit evidence from research or other sources directly, but relied on ‘mindlines’ – collectively reinforced, internalised, tacit guidelines. These were informed by brief reading but mainly by their own and their colleagues’ experience, their interactions with each other and with opinion leaders, patients, and pharmaceutical representatives, and other sources of largely tacit knowledge” [23]. However, Gabbay and May did not justify their arguments on clinical data as has been done in our case.

To facilitate a more rational prescription, the factors underlying that variability should be assessed, such as the characteristics of health professionals and patients, safety, as well as the effectiveness and costs of the analysed drugs. 

In the case of SYSADOA, in the Basque Country, the reasons possibly underlying the variability were explored through qualitative research, including a focus group in primary care, and individual interviews with specialized doctors in the region that showed higher prescription rates [24]. We found that the prescription of SYSADOA was multifactorial, and could be influenced by the lack of knowledge around the quality of the evidence; inadequate referral to specialized care; induced prescription; the demand from patients; and the lack of interdisciplinary consensus [24].

Possible limitations of our study are that we used prescription data obtained from drugstores, which means that:

1. Billing for prescriptions reliably refers to the dispensation of drugs, but not necessarily to what the doctors have prescribed or what patients have consumed.
2. It is not possible to link the prescription with the information from the patient clinical record (diagnostic, therapeutic indication) directly, which implies that the variability cannot be interpreted in terms of adequacy.

However, this is not relevant in the case of SYSADOA, since they are only indicated and used for the treatment of osteoarthritis. 

This is the first publication of a larger project which aimed to discuss if any changes could be made in the SYSADOA prescription routine of PC physicians for the management of osteoarthritis.

Another limitation concerns the DDD. While the ATC/DDD system allows for comparative studies between different populations and therapeutic groups, the practice of increasing doses due to clinical strategies for certain values of some physiological parameters with regard to the management of particular risk factors (such as hypertension), might result in a DDD significantly higher than the usual dose. In addition, in many cases combined treatments (same patients total DDD that receive two or more drugs) are used. For both reasons, the DDD tends to overestimate the number of people actually treated. This is not the case in our study. Since 2011, a combination has been available, and duplicated active ingredients in the Basque Health Region could be detected by the electronic software that controls prescriptions.

The analysis of variability performed in this study reflects the use of the different SYSADOA. Even with the limitations derived from having obtained the data from databases of billing prescriptions without any information on the diagnosis, this type of population analysis could be used to identify prescription rates and monitor the effectiveness of the implemented pharmaceutical policies.

We found that similar situations occurred in other high-income countries. In the case of South Korea, in a report published in 2009 by the National Evidence-Based Healthcare Collaborating Agency (NECA), it was estimated that, of the population of 40 years or over, 12.18% were using glucosamine daily, and 29.93% had been prescribed glucosamine at some point [25]. The total spending on products with glucosamine was estimated at 280 trillion Korean won or 232 million euros per year (6,000 won per person, approximately 5 euros per person) [26]. However, the authors pointed out that although the results of this report supported a reduced intake of glucosamine, the effect on policy decisions was limited [26]. This observed prescription practice is similar to that obtained in our study, so data are consistent for two aging populations even geographically and ethnically separated.

In 2010 and 2011, other countries, such as Sweden or Denmark, decided not to reimburse these drugs due to lack of evidence of their efficacy [27], [28].

In the case of Spain, the pharmaceutical reimbursement policies of drugs in outpatient care are established at the national level, and regions could only suggest or modulate prescription through the use of evidence-based prescription audits and feedbacks. This shows that in order to control the prescription of these drugs adequately, an interdisciplinary evidence-based dialogue is necessary, establishing agreed management criteria with the patient that favoured the de-prescription of the SYSADOA. However, in addition to these controls, patients should consistently be given information on what they could do to improve their clinical condition.

## Conclusions

Despite the uncertainty around their efficacy, SYSADOA are still being prescribed in the Basque Country. Several countries established policies on the reimbursement of these drugs and even removed them from public reimbursement. Nevertheless, in the Basque Country (and also in Spain), these drugs continue to be prescribed and reimbursed. The uncertainty with regard to evidence could be one of the reasons for variability in clinical practice. 

Although Spain is a decentralized country, public policies regarding reimbursement are defined at the national level. However, the variability found in our study could lead to tailored strategies for disinvestment in which those regions with higher prescription rates will be prioritized for disinvestment activities. These policies should ideally be discussed with professionals and patients to ensure their implementation in clinical practice.

## Notes

### Competing interests

The authors declare that they have no competing interests.

### Acknowledgements

The authors would like to thank the members of the Pharmacy Directorate of the Ministry for Health of the Basque Country and the Interior Primary Care Setting for providing the data and thus allowing for its analysis. 

### Funding

The authors received a grant from the Basque Center for Health Services, Research and Chronicity (KRONIKGUNE); grant number: kronik11/037. The funding body did not influence the design of the study, the collection, analysis, and interpretation of the data, or the writing of the manuscript. 

## Figures and Tables

**Table 1:**
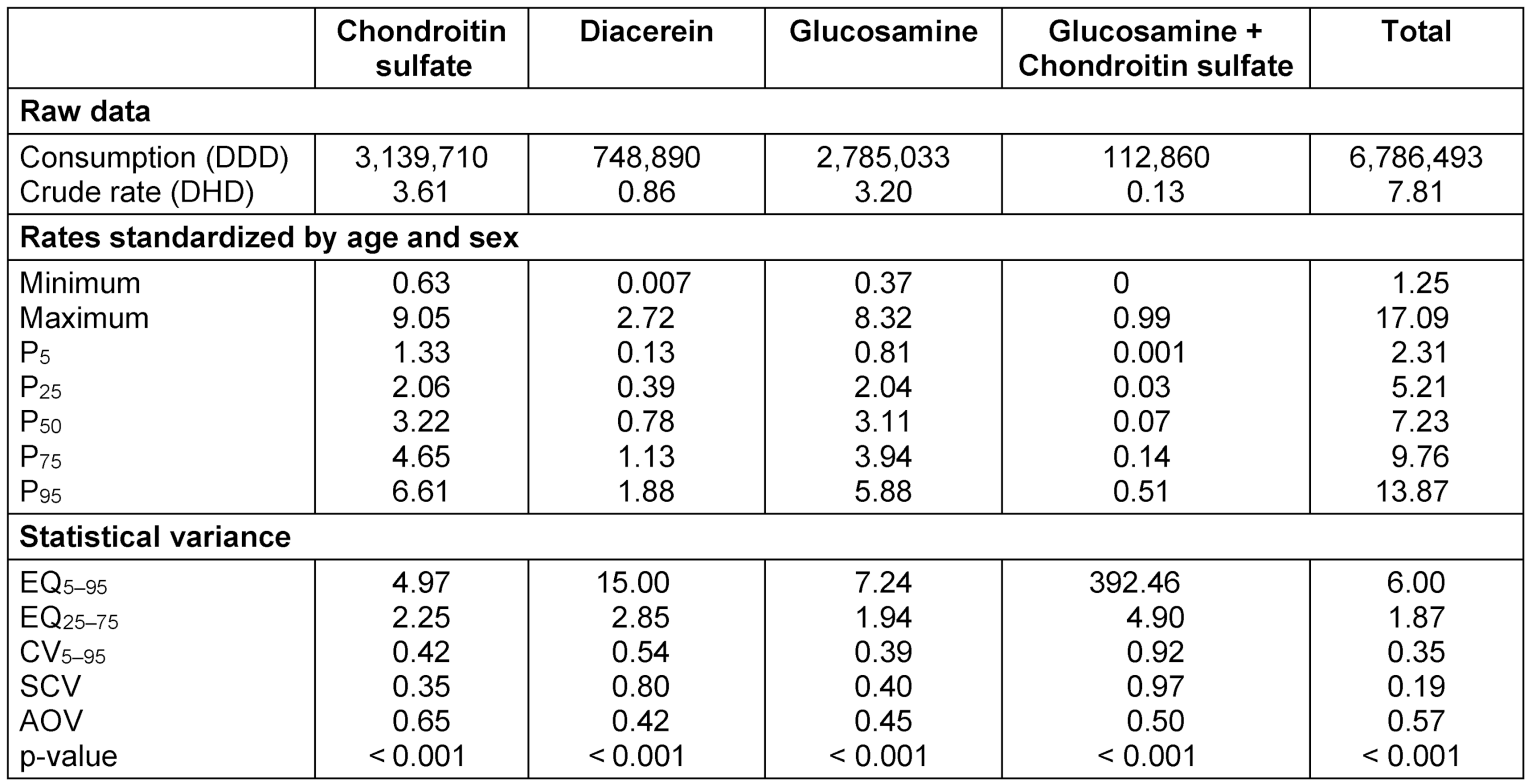
Raw and standardized rates by 1,000 inhabitants and day and statistics of variation. P: percentile; EQ: extreme quotient; CV: coefficient of variation; SCV: systematic component of the variation; AOV: variance explained by the factor region in the analysis of the variance. The subscript indicates that the parameter has been calculated considering those areas whose standardized rate is among the percentiles indicated.
